# Type I and III Interferon Productions Are Impaired in X-Linked Agammaglobulinemia Patients Toward Poliovirus but Not Influenza Virus

**DOI:** 10.3389/fimmu.2018.01826

**Published:** 2018-08-10

**Authors:** Anderson Dik Wai Luk, Ke Ni, Yuet Wu, Kwok-Tai Lam, Koon-Wing Chan, Pamela P. Lee, Wenwei Tu, Huawei Mao, Yu Lung Lau

**Affiliations:** ^1^Department of Paediatrics and Adolescent Medicine, LKS Faculty of Medicine, The University of Hong Kong, Hong Kong, Hong Kong; ^2^Institute of Biology, Westlake Institute for Advanced Study, Hangzhou, Zhejiang, China; ^3^Shenzhen Primary Immunodeficiency Diagnostic and Therapeutic Laboratory, Department of Paediatrics, The University of Hong Kong-Shenzhen Hospital, Shenzhen, China; ^4^Department of Rheumatology and Immunology, Ministry of Education Key Laboratory of Child Development and Disorder, Children’s Hospital of Chongqing Medical University, Chongqing, China

**Keywords:** type I interferon, type III interferon, X-linked agammaglobulinemia, oral poliovirus vaccine, H1N1 influenza virus, innate immunity, dendritic cells

## Abstract

**Background:**

X-linked agammaglobulinemia (XLA) is a primary immunodeficiency caused by Bruton’s tyrosine kinase (*BTK*) mutation. Patients are susceptible to severe enterovirus infections. The underlying mechanism remains unknown. BTK is involved in toll-like receptors pathway, which initiates antiviral responses including interferon (IFN) productions.

**Objective:**

To demonstrate type I and III IFN productions in dendritic cells of XLA patients is decreased in response to oral poliovirus vaccine (OPV) but not H1N1 virus.

**Methods:**

Monocyte-derived dendritic cells (MoDCs) were derived from nine XLA patients aged 22–32 years old and 23 buffy coats from Hong Kong Red Cross blood donors. LFM-A13 was used to inhibit BTK. OPV Sabin type 1 and H1N1 influenza virus were used to stimulate MoDCs with RPMI as mock stimulation. The antiviral cytokine productions and phenotypic maturation of MoDCs were determined 24 h post-stimulation. OPV RNA was determined at 0, 6, 12, and 24 h post-stimulation.

**Results:**

Upon OPV stimulation, IFN-α2, IFN-β, and IFN-λ1 productions in MoDCs from XLA patients and BTK-inhibited MoDCs of healthy controls were significantly lower than that from healthy controls. Whereas upon H1N1 stimulation, the IFN-α2, IFN-β, and IFN-λ1 productions were similar in MoDCs from XLA patients, BTK-inhibited MoDCs of healthy controls and healthy controls. The mean fluorescent intensities (MFI) of CD83, CD86, and MHC-II in MoDCs from XLA patients in response to OPV was similar to that in response to mock stimulation, while the MFI of CD83, CD86, and MHC-II were significantly higher in response to H1N1 stimulation than that in response to mock stimulation. Whereas, the MFI of CD83, CD86, and MHC-II in MoDCs of healthy controls were significantly higher in response to both OPV and H1N1 stimulation compared to that in response to mock stimulation.

**Conclusion:**

Production of type I and III IFN in response to OPV was deficient in MoDCs from XLA patients, but was normal in response to H1N1 due to deficient BTK function. Moreover, phenotypic maturation of MoDCs from XLA patients was impaired in response to OPV but not to H1N1. These selective impairments may account for the unique susceptibility of XLA patients toward severe enterovirus infections.

## Introduction

X-linked agammaglobulinemia (XLA) is a primary immunodeficiency caused by mutations of Bruton’s tyrosine kinase (*BTK*) (1, 2). BTK is expressed in all lineages of hematopoietic cells except T cells (3). As a result, XLA patients typically suffer from recurrent respiratory infections caused by encapsulated bacteria but are generally not susceptible to viral infections (1, 4). However, XLA patients are particularly vulnerable to severe enterovirus infections, notably chronic meningoencephalitis from Echo virus and vaccine-associated paralytic poliomyelitis (VAPP) from live-attenuated oral poliovirus vaccine (OPV) (5–9). Antibodies deficiency has been suggested to be responsible for the increase in susceptibility (10), but enterovirus infections in XLA patients with adequate immunoglobulin replacement have been reported (11, 12), indicating that antibody deficiency alone cannot fully explain the susceptibility toward severe enterovirus infections. To date the exact mechanism behind this unique susceptibility is unknown.

Enteroviruses are mainly transmitted through feco-oral route. The viruses then replicate in the upper respiratory tract, distal small bowel, and submucosal lymphoid tissues, leading to viremia and sometimes disseminated infections in the central nervous system (CNS), heart, and skin (7). Innate immunity, particularly interferons (IFN), as well as T cell-mediated immune responses are critical in protecting hosts from enterovirus infections (13).

Type I and III IFN play important roles in mucosal immunity and are often among the first cytokines produced in response to viral infections, restricting viral replications. Majority of the antiviral actions of type I IFN are exerted through the expression of interferon-stimulated genes (ISGs) (14, 15). ISGs are heterogeneous proteins with myriad antiviral mechanisms that target the different stages of viral life cycles (16), from blocking nuclear import of viral nucleocapsids by MX dynamin like GTPase 1 [MX dynamin like GTPase 1 (MX1)] to inhibiting translations by 2′-5′ oligoadenylate synthetase (OAS) and protein kinase R (PKR) (14, 17, 18). Production of ISGs such as interferon regulatory factor 3 (IRF3), IRF7, and toll-like receptor 3 (TLR3) sensitize pathogen detection and enhance IFN production to amplify antiviral responses (14). In addition, type I IFN has a broad range of effects on innate and adaptive immune cells that eventually result in clearance of viral infections (14). Type III IFN has similar effects as type I IFN but its actions are restricted to mucosal epithelium, liver, and a handful of immune cells including plasmacytoid dendritic cells (pDCs) and monocyte-derived macrophages (15).

Innate immune cells sense pathogens by recognizing the pathogen-associated molecular patterns (PAMPs) *via* the pattern recognition receptors (PRRs) (14, 15). Toll-like receptors (TLRs) and retinoic acid-inducible gene I (RIG)-I-like receptors (RLRs) are the two main PRRs to detect viral infections. TLR3, 7, 8, 9, and melanoma differentiation-associated protein 5 (MDA5) are utilized to detect enterovirus infections (2, 19) while TLR3, 7, 8, and RIG-I are utilized to detect influenza virus infections (20).

**Figure 1:**
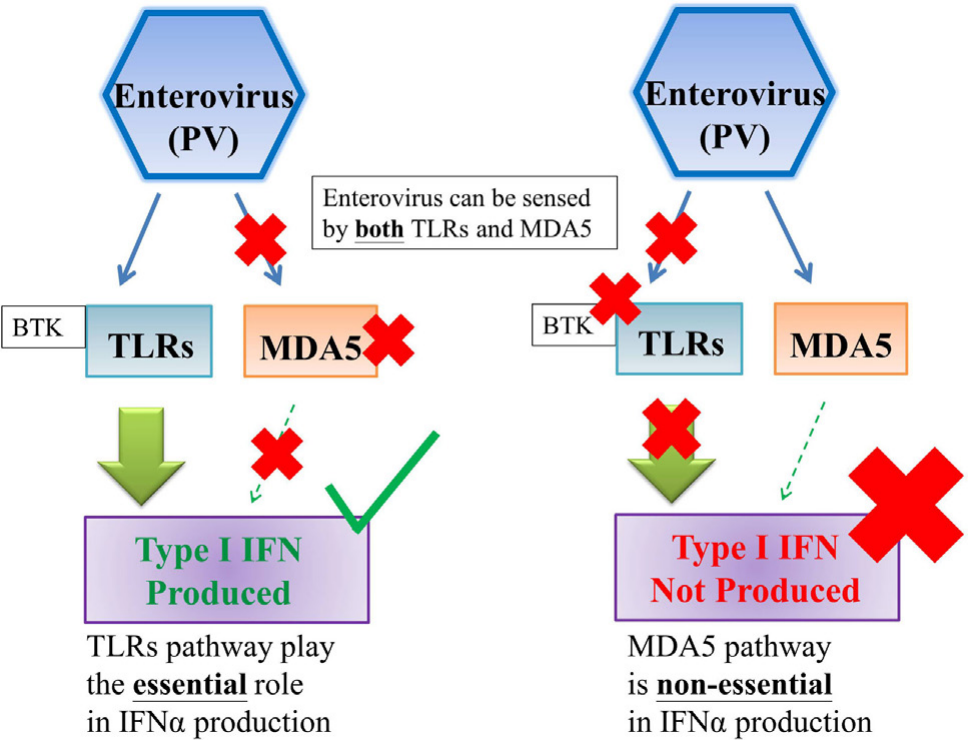
TLRs pathway, but not MDA5 pathway, is essential to the production of type I interferon against enterovirus infections. Enterovirus can be sensed by both TLRs and MDA5; however, TLRs pathway, but not MDA5 pathway, plays the essential role on type I interferon production against enterovirus infections (2). Abbreviations: PV, poliovirus; TLRs, toll-like receptors; MDA5, melanoma differentiation-associated protein 5; IFN, interferon.

**Figure 2:**
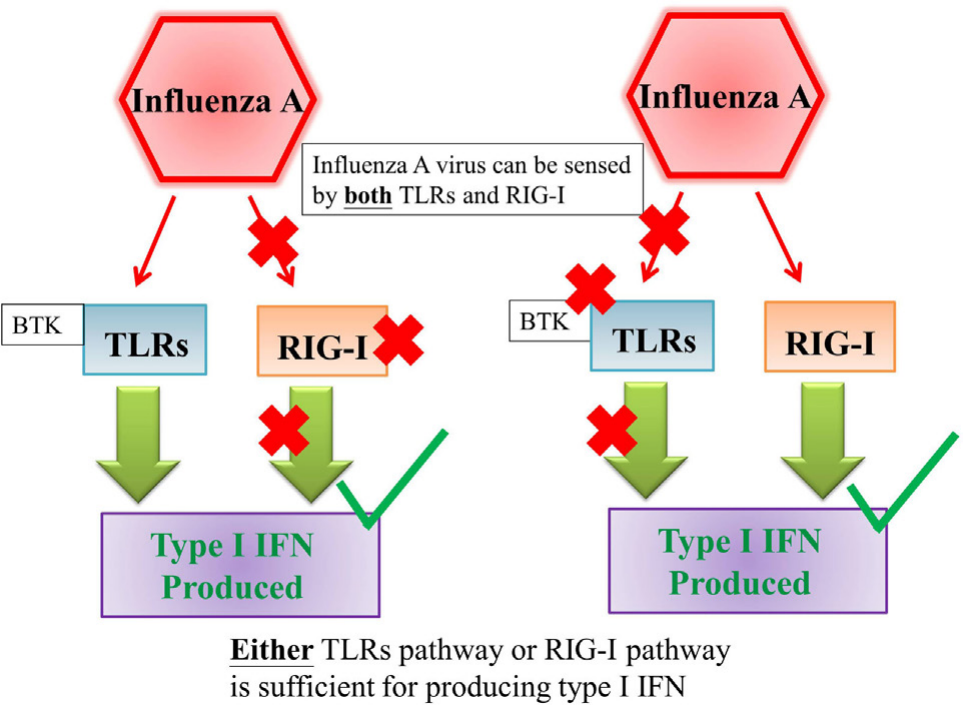
Either TLRs pathway or RIG-I pathway is sufficient for producing type I interferon against influenza A virus infection. Influenza A virus can be sensed by both TLRs and RIG-I and either TLRs pathway or RIG-I pathway is sufficient for producing type I interferon against influenza A virus infection (22). Abbreviations: TLRs, toll-like receptors; RIG-I, retinoic acid-inducible gene I; IFN, interferon.

Activation of PRRs by viral PAMPs triggers downstream signaling cascades which lead to the production of type I and III IFN, along with other cytokines (14, 15). The influence of the TLRs and RLRs pathways on initiating IFNs productions varies from virus to virus (Figures 1 and 2). It has been shown that the TLR pathways, but not the MDA5 pathway, play the essential role in initiating IFN productions in response to enterovirus infections (Figure 1) (2, 21). On the other hand, it has been shown either TLRs or RLRs on its own is sufficient to initiate the production of type I IFN in response to influenza virus infection (Figure 2) (22).

Recent studies have revealed specific roles of BTK in TLR signaling pathways, from directly phosphorylating the TLR (23) to interacting with the adapters of TLRs (24–27). We, therefore, hypothesized that XLA patients have impaired type I and III IFN productions in response to enteroviruses but not to other viruses in a BTK-dependent manner. In this study, we sought to demonstrate type I and III IFN productions are decreased in response to OPV, but normal to H1N1 virus in monocyte-derived dendritic cells (MoDCs) of XLA patients.

## Materials and Methods

### Subjects

Nine XLA patients aged 22–32 years old were recruited for the study (Table 1). All of the nine patients have received OPV vaccination before and none had a history of acute flaccid paralysis before or excreting vaccine-derived poliovirus (VDPV). 40 mL of heparinized fresh blood was drawn for the study before the commencement of their regular intravenous immunoglobulin replacement therapy in Queen Mary Hospital. Twenty-three donor buffy coats from Hong Kong Red Cross were obtained as healthy control. This study was approved by the Institutional Review Board of the University of Hong Kong/Hospital Authority Hong Kong West Cluster (UW 08-002). All subjects gave written informed consent in accordance with the Declaration of Helsinki.

**Table 1:** Bruton’s tyrosine kinase mutations of the nine XLA patients.

Patient	Age	Exon/Intron	cDNA change involved	Amino acid change	Protein domain
P001a	26	E2	c.41C > A	S14Y	PH domain
P001b	27	E2	c.41C > A	S14Y	PH domain
P002	23	E11	c.942A > G	G313fsX318	SH2 domain
P003	22	E14	c.1278delC	D426fsX431	PK domain
P004	32	E2	c.3G > T	M1I	(Start codon)
P005	28	E2	c.3G > T	M1I	(Start codon)
P006	30	E10	c.885_887delA	K296fsX330	SH2 domain
P007	25	E2	c.3G > T	M1I	(Start codon)
P008	24	I15	g.IVS15-2A > T, c.1567-1631del	A523fsX527	PK domain

*PH, pleckstrin homology; SH2, Src homology 2; PK, protein kinase; XLA, X-linked agammaglobulinemia. All nine patients received oral poliovirus vaccine and had no history of acute flaccid paralysis and no excretion of vaccine-derived poliovirus. P001a and P001b were from the same kindred*.

### Generation of Monocyte-Derived Dendritic Cells

Monocytes of patients and healthy controls were obtained from peripheral blood mononuclear cells (PBMC) using CD14 MicroBeads (Miltenyi Biotec, Germany), and were cultured in RPMI with 10% fetal bovine serum (FBS), 50 ng/mL GM-CSF, and 10 ng/mL IL-4 for 6 days to obtain monocyte-derived dendritic cells (MoDCs) as described previously (4).

Bruton’s tyrosine kinase-inhibited MoDCs were generated by pretreating MoDCs from healthy control with LFM-A13 (Calbiochem, USA) at 150 µM (Figure S1 in Supplementary Material) 2 h prior to viral stimulation (23, 28).

### Viral Stimulation of MoDCs

In our previous study, we have optimized the conditions of stimulating MoDCs of XLA patients with H1N1 (4). In our current study, OPV Sabine type 1 and influenza A virus H1N1 were used to stimulate MoDCs on day 6. H1N1 virus was incubated at 56°C for 30 min before used to stimulate MoDCs as described previously (4). RPMI was used as mock stimulation. MoDCs and BTK-inhibited MoDCs from healthy controls, and MoDCs from XLA patients were incubated with OPV and H1N1 at multiplicity of infection (MOI) of 1 in the absence of FBS at 37°C for 2 h. 10% FBS was then supplemented to MoDCs.

All procedures involving viruses were conducted in biosafety level 2 (BSL 2) laboratory and in accordance with the World Health Organization polio laboratory manual (29).

### Phenotypic and Cytokine Analysis

At 24 h post-stimulation, supernatant and MoDCs were collected for analysis. Antiviral cytokines levels in the supernatant were measured using cytometric bead array [LEGENDplex™ Human Anti-Virus Response Panel (13-plex), BioLegend, San Diego, CA, USA].

Phenotypic maturation of MoDCs was analyzed by flow cytometry using anti-CD14 PB, anti-CD86 PE, anti-MHC II FITC, anti-CD83 FITC, and anti-CD155 APC (Biolegend, San Diego, CA, USA).

### RNA Analysis

Viral RNA of OPV was determined at 0, 6, 12, and 24 h post-stimulation in MoDCs from healthy controls and patients. *IRF3, IRF7, TLR3, PKR, MX1, OAS1*, and *IFN*-α*2* RNA were determined at 0, 24, and 48 h post-stimulation in MoDCs from healthy controls and XLA patients by OPV.

Total RNA was extracted from MoDCs and supernatant using TaKaRa MiniBEST Universal RNA Extraction Kit (TaKaRa, Japan). cDNA conversion was performed using TaKaRa PrimeScript RT reagent Kit (TaKaRa, Japan). Quantitative PCR for OPV (Custom TaqMan^®^ Gene Expression Assay PN4331348, Assay ID: AIY9Z0P, ThermoFisher, USA), *IRF3* (TaqMan^®^ Gene Expression Assay 4331182, Assay ID: Hs01547283_m1), *IRF7* (TaqMan^®^ Gene Expression Assay 4331182, Assay ID: Hs00185375_m1), *TLR3* (TaqMan^®^ Gene Expression Assay 4331182, Assay ID: Hs00152933_m1), *PKR* (TaqMan^®^ Gene Expression Assay 4331182, Assay ID: Hs00169345_m1), *MX1* (TaqMan^®^ Gene Expression Assay 4331182, Assay ID: Hs00182073_m1), *OAS1* (TaqMan^®^ Gene Expression Assay 4331182, Assay ID: Hs00973635_m1), and *IFN*- α*2* (TaqMan^®^ Gene Expression Assay 4331182, Assay ID: Hs00265051_s1) was performed using ABI 7900 sequence detection system (Applied Biosystems). The amplification was performed with denaturation for 20 s at 95°C followed by 40 cycles of 95°C for 2 s and 60°C for 30 s. β-*actin* (Hs99999903_m1, TaqMan Gene Expression Assays, ThermoFisher, USA) and glyceraldehyde-3-phosphate dehydrogenase (*GAPDH*) (Hs02758991_g1, TaqMan Gene Expression Assays, ThermoFisher, USA) were used as internal control. Arbitrary threshold cycle of 40 was set for measurements under the detection limit of qPCR. Results were normalized to β-*actin* or *GAPDH* expression and presented as fold increase in RNA expression at 6, 12, 24, and 48 h post-stimulation compared to that at 0 h using the comparative threshold cycle method (ΔΔCt).

### Statistics

All data were expressed in mean ± SEM. Statistics were analyzed by one-way ANOVA with Dunnett’s multiple comparison test, unpaired *t*-test, or Wilcoxon matched-pairs signed rank test using Prism 7 (GraphPad Software). Statistical significance was defined as *p* < 0.05.

## Results

### Production of IFN-α2, IFN-β, and IFN-λ1 Were Impaired in a BTK-Dependent Manner in MoDCs of XLA Patients Upon OPV Stimulation

Cytokines produced upon OPV stimulation from the MoDCs of XLA patients, BTK-inhibited MoDCs and MoDCs of healthy controls were measured at 24 h post-stimulation.

**Figure 3:**
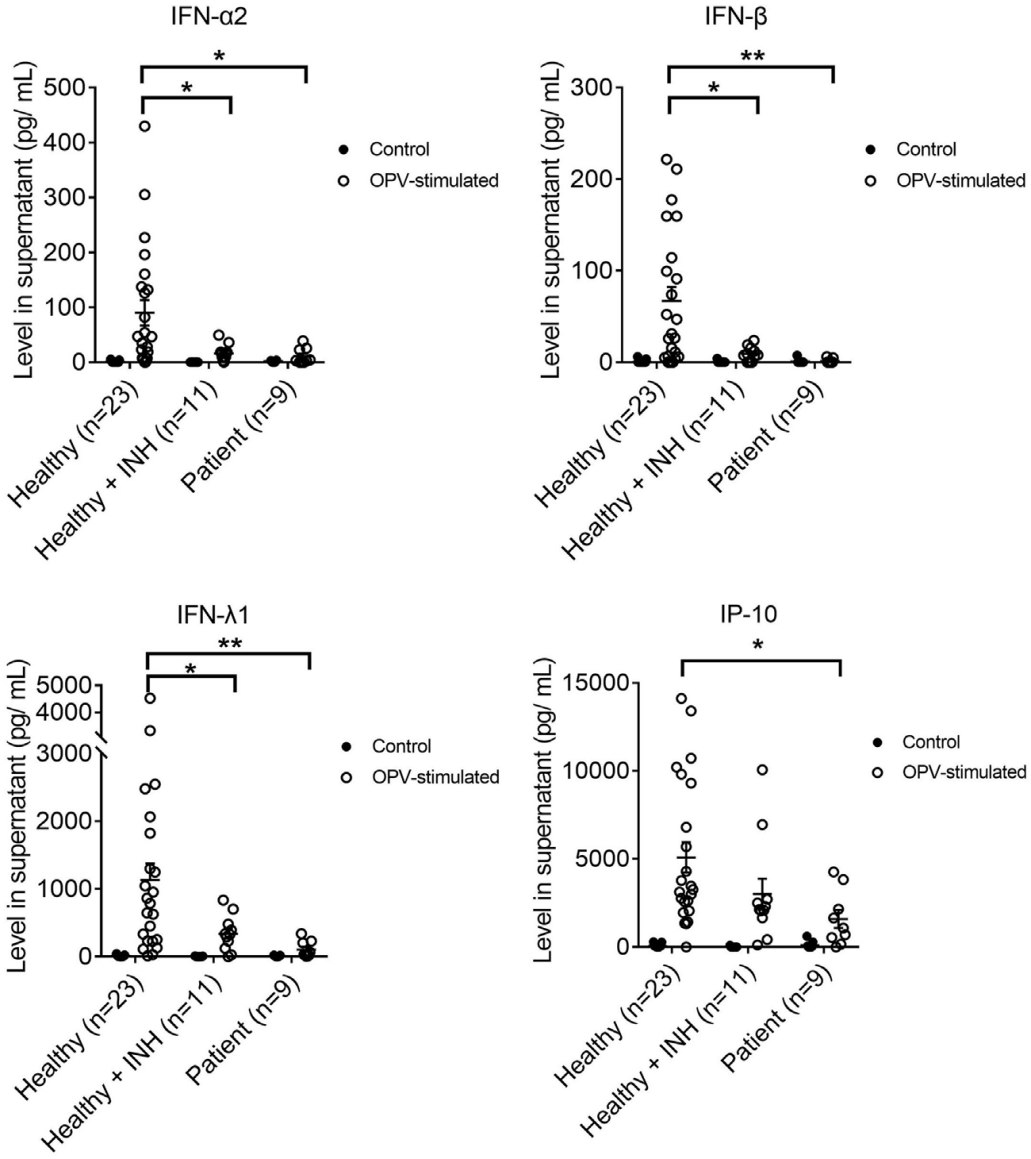
IFN-α2, IFN-β, IFN-λ1, and interferon gamma-induced protein 10 productions in MoDCs from healthy controls (*n* = 23), Bruton’s tyrosine kinase (BTK)-inhibited MoDCs from healthy controls (*n* = 11), and MoDCs from X-linked agammaglobulinemia patients (*n* = 9) upon OPV stimulation. MoDCs were stimulated with OPV at multiplicity of infection of 1 for 24 h. Open symbols represent MoDCs stimulated with OPV; filled symbols represent MoDCs that were mock-stimulated with RPMI. Healthy + INH, BTK-inhibited MoDCs from healthy controls. Data represented as mean ± SEM. *p < 0.05 and **p < 0.01.

IFN-α2, IFN-β, IFN-λ1, and interferon gamma-induced protein 10 (IP-10) productions were lower in MoDCs from XLA patients compared to that from healthy controls upon OPV stimulation (Figure 3).

IFN-α2, IFN-β, and IFN-λ1 productions were lower in BTK-inhibited MoDCs of healthy controls compared to that from MoDCs of healthy controls upon OPV stimulation (Figure 3).

There was no difference in the production of IFN-α2, IFN-β, IFN-λ1, and IP-10 between MoDCs from patients and healthy controls upon mock stimulation (Figure 3). There was no difference in the production of interleukin-1 beta (IL-1β), tumor necrosis factor alpha (TNF-α), and IL-8 between MoDCs from patients and healthy control upon OPV stimulation (Figure S2 in Supplementary Material).

### Production of IFN-α2, IFN-β, and IFN-λ1 Were Normal in MoDCs of XLA Patients Upon H1N1 Stimulation

Cytokines produced upon H1N1 stimulation from the MoDCs of XLA patients, BTK-inhibited MoDCs and MoDCs of healthy controls were measured at 24 h post-stimulation.

**Figure 4:**
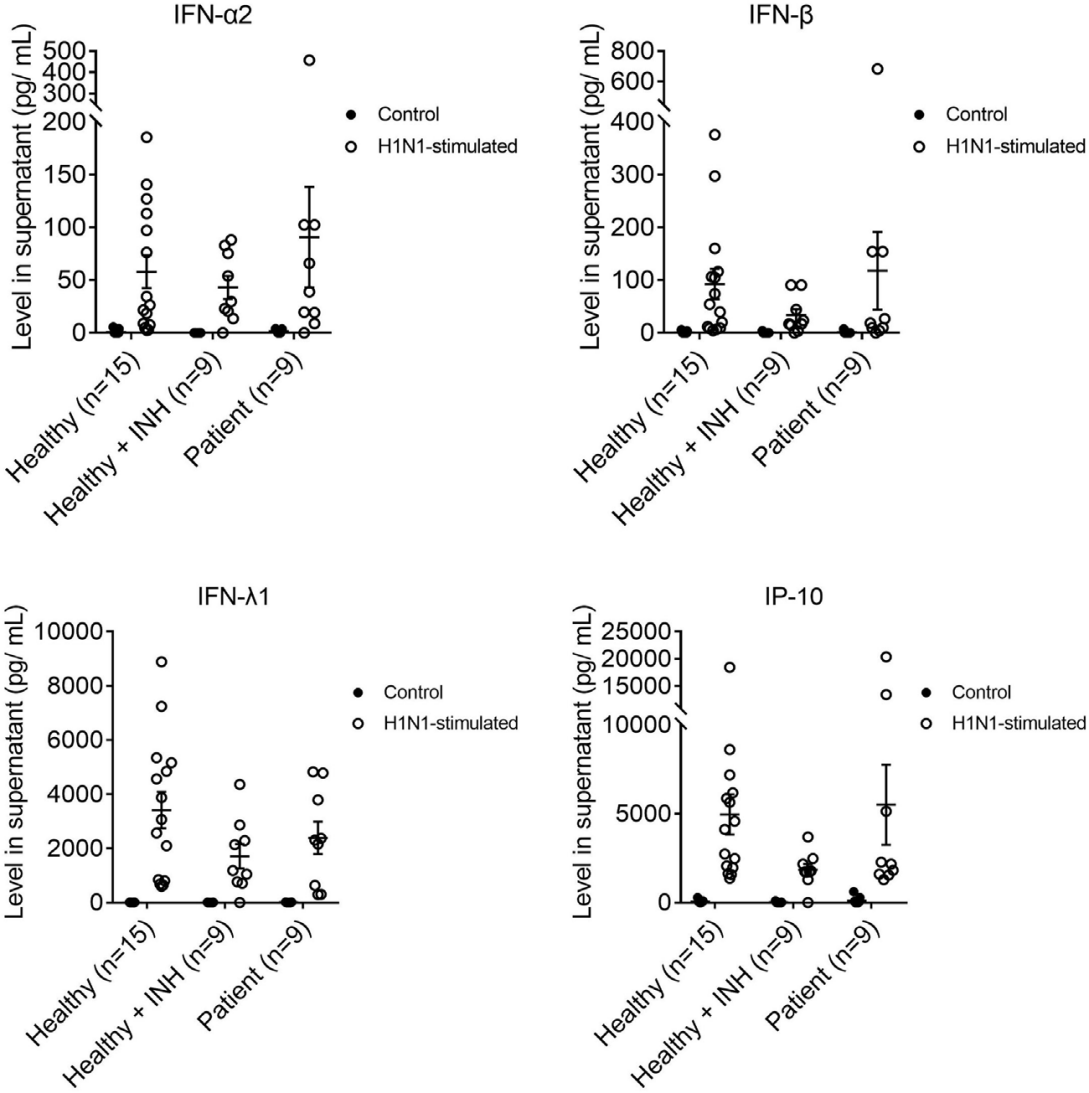
IFN-α2, IFN-β, IFN-λ1, and interferon gamma-induced protein 10 productions in MoDCs from healthy controls (*n* = 15), Bruton’s tyrosine kinase (BTK)-inhibited MoDCs from healthy controls (*n* = 9), and MoDCs from X-linked agammaglobulinemia (XLA) patients (*n* = 9) upon H1N1 stimulation. MoDCs were stimulated with H1N1 at multiplicity of infection of 1 for 24 h. Open symbols represent MoDCs stimulated with H1N1; filled symbols represent MoDCs that were mock-stimulated with RPMI. Controls of healthy controls, BTK-inhibited healthy controls, and XLA patients were subsets of controls in Figure 3. Healthy + INH, BTK-inhibited MoDCs from healthy controls. Data represented as mean ± SEM.

There was no difference in IFN-α2, IFN-β, IFN-λ1, and IP-10 productions among the MoDCs of XLA patients, BTK-inhibited MoDCs, and MoDCs of healthy controls upon H1N1 stimulation (Figure 4). There was no difference in the production of IL-1β, TNF-α, and IL-8 among the three groups upon H1N1 stimulation (Figure S3 in Supplementary Material).

### Expression of *IRF7, TLR3, PKR, MX1, OAS1*, and *IFN*-α*2* Were Impaired in XLA Patients Following OPV Stimulation

RNA expressions of *IRF3, IRF7, TLR3, PKR, MX1, OAS1*, and *IFN*-α*2* in MoDCs of XLA patients and healthy controls were measured at 24 and 48 h post-stimulation with OPV.

**Figure 5:**
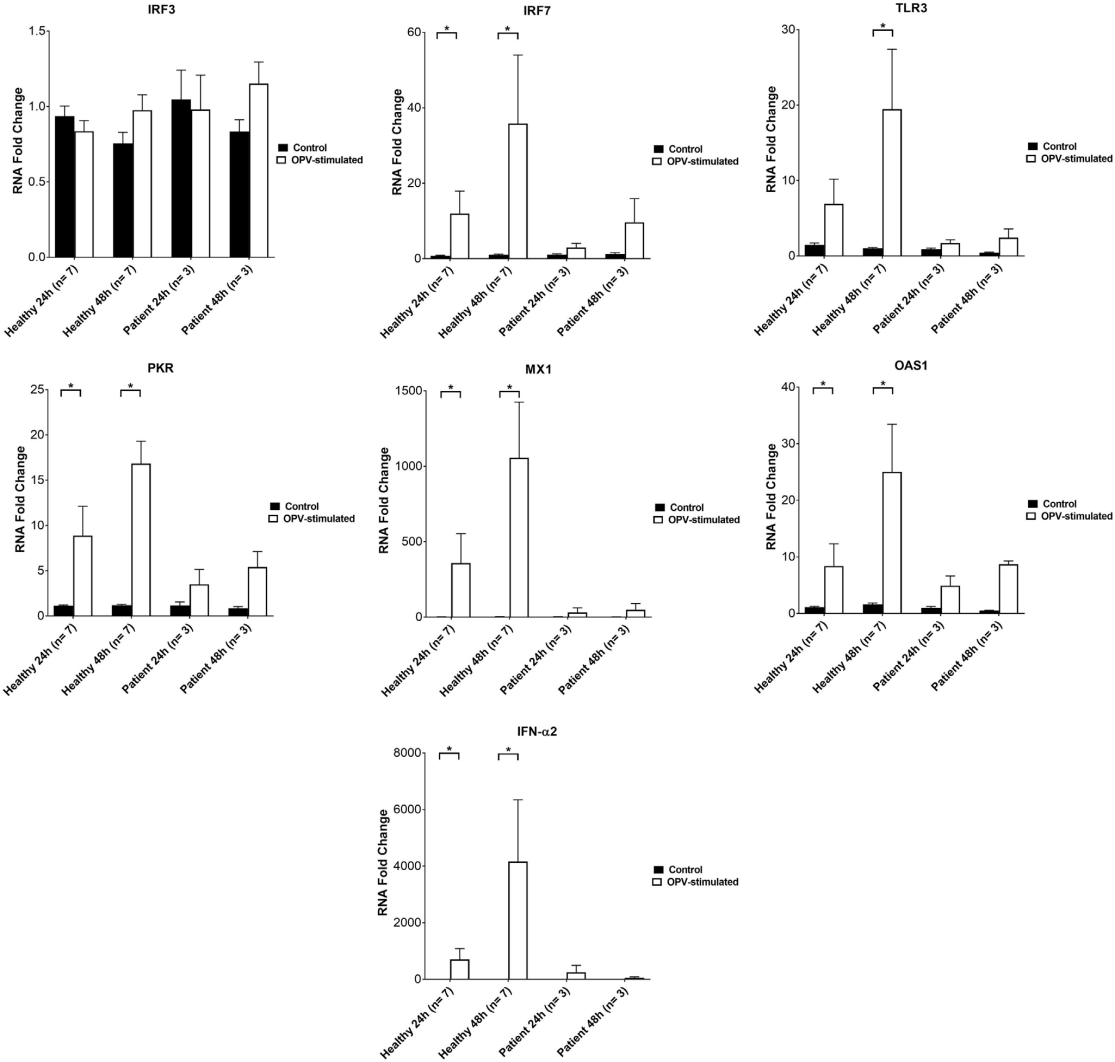
*IRF3, IRF7, TLR3, PKR, MX1, OAS*, and *IFN*-α*2* RNA levels in MoDCs from healthy controls (*n* = 7) and X-linked agammaglobulinemia patients (*n* = 3) after 0 to 48 h post-infection. MoDCs were treated with OPV at multiplicity of infection of 1. Open bars represent RNA fold changes in MoDCs treated with OPV; filled bars represent RNA fold changes in MoDCs treated with RPMI. Fold change of RNA at 24 and 48 h compared to 0 h post-infection was calculated by the “ΔΔCt” method using GAPDH as endogenous control. Abbreviations: IRF, interferon regulatory factor; TLR, toll-like receptor; PKR, protein kinase R; MX1, MX dynamin-like GTPase 1; OAS1, 2′-5′ oligoadenylate synthetase 1; IFN, interferon; GAPDH, glyceraldehyde-3-phosphate dehydrogenase. Data represented as mean ± SEM. Statistics were analyzed by Wilcoxon matched-pairs signed rank test. *p < 0.05.

There was no increase in RNA expressions of *IRF3, IRF7, TLR3, PKR, MX1, OAS1*, and *IFN*-α*2* in MoDCs of XLA patients upon OPV stimulation (Figure 5).

RNA expressions of *IRF7, PKR, MX1, OAS1*, and *IFN*-α*2* were increased in MoDCs of healthy controls at 24 and 48 h post-stimulation with OPV (Figure 5). RNA expression of *TLR3* was increased in MoDCs of healthy control at 48 h post-stimulation with OPV (Figure 5).

There was no increase in RNA expression of *IRF3* in MoDCs of healthy control upon OPV stimulation (Figure 5).

### Phenotypic Maturation of XLA Patients DC Was Impaired Upon OPV but Not H1N1 Stimulation

MoDCs from patients, BTK-inhibited MoDCs, and MoDCs of healthy controls were collected at 24 h post-stimulation with OPV and H1N1 for flow cytometry.

**Figure 6:**
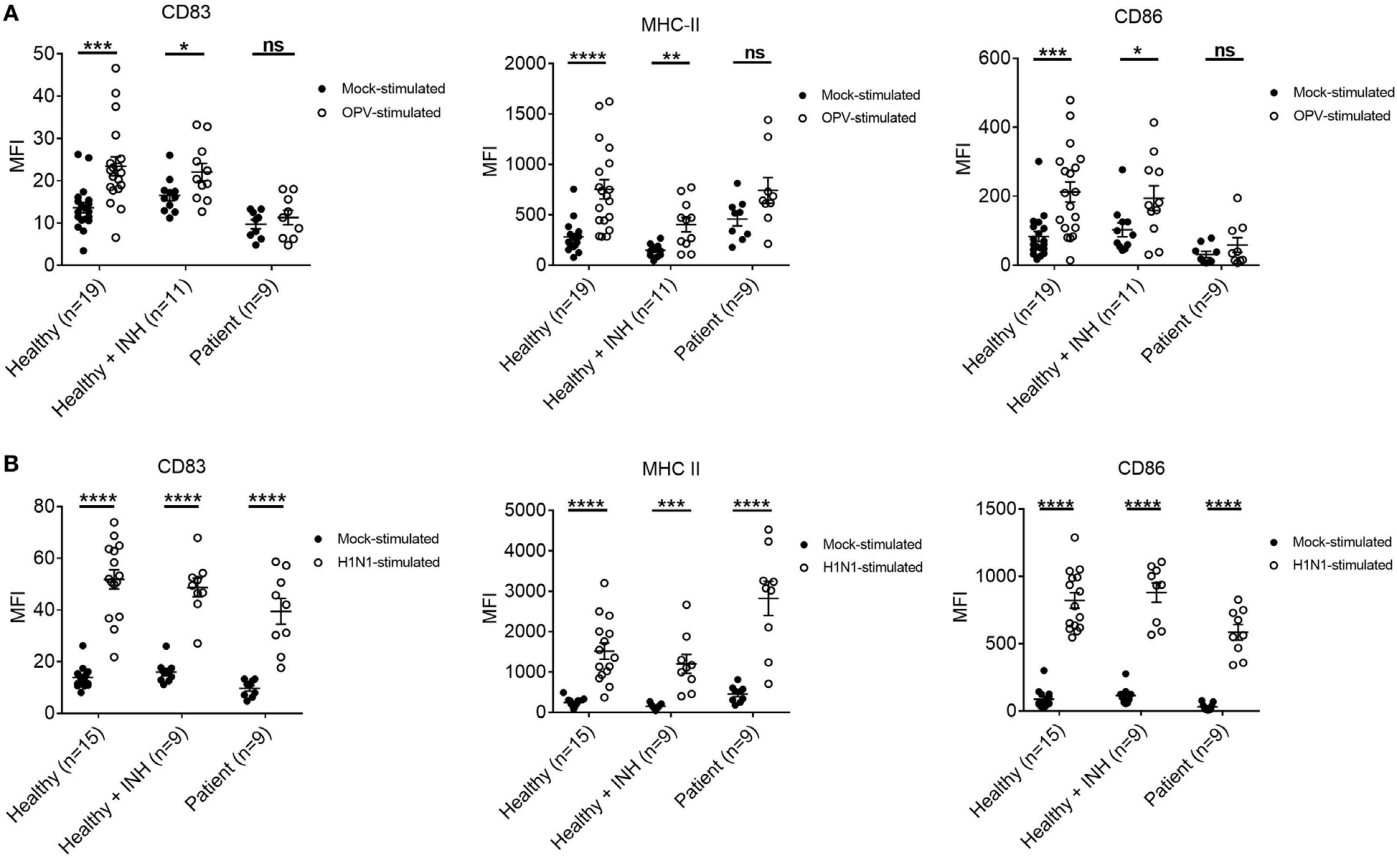
Surface marker changes in MoDCs from healthy controls, Bruton’s tyrosine kinase (BTK)-inhibited MoDCs from healthy controls, and MoDCs from X-linked agammaglobulinemia (XLA) patients upon OPV and H1N1 stimulation. MoDCs were stimulated with OPV and H1N1 at multiplicity of infection of 1 for 24 h. Open symbols represent MoDCs stimulated with OPV or H1N1; filled symbols represent MoDCs that were mock-stimulated with RPMI. Healthy + INH, BTK-inhibited MoDCs from healthy controls. Data represented as mean ± SEM. ns, *p* > 0.05; *p < 0.05; **p < 0.01; ***p < 0.001; and ****p < 0.0001. **(A)** MFI of CD83, MHC-II, and CD86 in MoDCs from healthy controls (*n* = 19), BTK-inhibited MoDCs from healthy controls (*n* = 11), and MoDCs from XLA patients (*n* = 9) upon OPV stimulation. **(B)** MFI of CD83, MHC-II, and CD86 in MoDCs from healthy controls (*n* = 15), BTK-inhibited MoDCs from healthy controls (*n* = 9), and MoDCs from XLA patients (*n* = 9) upon H1N1 stimulation. Controls of MoDCs from healthy controls, BTK-inhibited MoDCs from healthy controls, and MoDCs from XLA patients were subsets of controls in Figure 6A.

Mean fluorescent intensity (MFI) of CD83, MHC-II, and CD86 of MoDCs and BTK-inhibited MoDCs of healthy controls were higher upon both OPV and H1N1 stimulation when compared to mock stimulation (Figure 6).

There was no difference in MFI of CD83, MHC-II, and CD86 of MoDCs from XLA patients upon OPV stimulation when compared to mock stimulation (Figure 6A).

However, MFI of CD83, MHC-II, and CD86 were higher in MoDCs from XLA patients upon H1N1 stimulation when compared to mock stimulation. Interestingly the MFI of MHC-II in MoDCs from patients was higher than that in MoDCs from healthy control (Figure 6B).

### No Difference of Viral Replication in MoDCs of XLA Patients and Healthy Control

**Figure 7:**
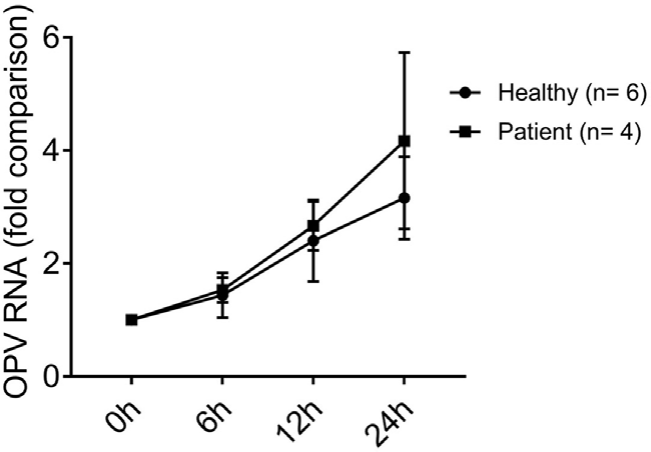
OPV RNA level in MoDCs from healthy controls and X-linked agammaglobulinemia (XLA) patients from 0 to 24 h post-infection. MoDCs from healthy controls (*n* = 6) and XLA patients (*n* = 4) were treated with OPV at multiplicity of infection of 1. Circles represent healthy control; squares represent patients. Fold change of viral RNA compared to 0 h post-infection was calculated by the “ΔΔCt” method using beta-actin as endogenous control. Data represented as mean ± SEM.

The fold increase of viral RNA at 6, 12, and 24 h were similar in MoDCs of XLA patients and healthy control (Figure 7).

## Discussion

Type I and III IFN productions were found to be near absent in MoDCs from XLA patients upon OPV stimulation. The deficits were replicated when BTK of healthy control MoDCs was inhibited, indicating the deficits were BTK-dependent. This is the first time such deficits were observed, which may be attributed to impairment of TLR signaling. Previous studies have shown that activation of TLR3, 4, 7, 8, and 9 can signal type I and III IFN productions (14, 15, 30). BTK has been shown to interact with the TLRs responsible for detecting OPV, phosphorylating the toll/interleukin-1 receptor (TIR) domain of TLR3 (23) as well as interacting with TIR domain of TLR 8 and 9 (24). In addition, BTK has been shown to interact with myeloid differentiation primary response gene 88, TIR-domain-containing adapter-inducing interferon-β, and TIR domain-containing adaptor protein (25, 26), which are the adapter molecules for TLRs. The impaired type I and III IFN responses in XLA patients could be due to interruption of TLR signaling by loss of BTK functions.

The near absence of type I and III IFN production in XLA patients impaired their antiviral responses toward OPV. MoDCs of XLA patients failed to express *PKR, MX1*, and *OAS1* in response to OPV (Figure 5), which are well-known ISGs to directly restrict viral replication and translation (14, 17, 18).

In addition to the above ISGs, MoDCs of XLA patients failed to up-regulate the expression of *IRF7* and *TLR3* in response to OPV, while OPV failed to induce *IRF3* expression in MoDCs of both XLA patients and healthy controls (Figure 5), possibly because IRF3 is constitutively expressed in all dendritic cell types and its expression cannot be further stimulated by IFN-α (31, 32). Apart from dendritic cells, previous study has also reported on the failure to induce *IRF3* expression in neuronal cells by OPV (33). IRF7 is not constitutively expressed in majority of the dendritic cell types except pDCs, but its expression can be strongly induced by type I IFN in MoDCs (34). IRF3 and IRF7 are integral components of the TLR signaling pathway for type I and III IFN production (14, 15). IRF3 is more important than IRF7 in inducing IFN-β (35, 36). On the other hand, IRF7 can induce a wider range of type I and III IFN (34–36) and is critical in inducing IFN-α (36). Furthermore, IRF7 is required to amplify IFN production to mount an adequate antiviral response through the following mechanism (37). Upon recognition of PAMPs, the constitutively expressed IRF3 is activated, rapidly produces a small amount of IFN-β, which then stimulates the expression of IRF7 to amplify and diversify the IFN production in an autocrine and a paracrine fashion (34, 35, 37). This positive feedback loop was absent in MoDCs of XLA patients, resulting in prolonged impairment of IFN production (Figure 5). Such impairment may impede the clearance of viral infection and may further increase the susceptibility of XLA patients toward OPV infection.

Interferon gamma-induced protein 10 production was found to be impaired in MoDCs from XLA patients in response to OPV stimulation, but such impairment was not demonstrated by inhibiting BTK in healthy controls. This may be due to the IP-10 production is regulated by other cytokines apart from type I IFN (38, 39), including TNF-α and IL-1 (40, 41), which were shown to be normal in XLA patients (Figure S2 in Supplementary Material). IP-10 is a chemokine attracting activated T lymphocytes, macrophages and natural killer (NK) cells (42). Impaired IP-10 production in XLA patients may result in impaired recruitment of T cells to attack enterocytes infected with OPV during vaccination, increasing the risk of developing complications (43).

Type I and III IFN productions were normal in MoDCs from XLA patients upon H1N1 stimulation, which was similar as reported previously (4). The IFN productions were normal even with the inhibition of BTK in healthy controls. This indicates XLA patients have a normal capacity to produce type I and III IFN, supporting the deficit of producing type I and III IFN in response to OPV resides in the difference in initiating IFN productions between enterovirus and influenza virus. OPV is sensed by TLRs and MDA5, which is a type of RLR (2), while H1N1 is sensed by TLRs and RIG-I (22). It has been shown that TLRs, but not MDA5, play the essential role in initiating IFN productions in response to poliovirus infection (Figure 1) (2, 21). On the other hand, it has been shown either TLRs or RLRs on its own is sufficient to initiate the production of type I IFN in response to influenza infection (Figure 2) (22). This difference in utilizing PRRs to initiate IFN productions between OPV and H1N1 maybe behind the selective type I and III IFN production impairments upon OPV but not H1N1 stimulation in XLA patients.

Production of IL-1β, IL-8, and TNF-α were not impaired in MoDCs of XLA patients upon OPV stimulation. Instead of using pathogens, many previous studies used TLR agonists as stimulants. To date, at least 19 such studies using TLR agonists have been performed to investigate the effect of BTK deficiency on cytokine productions (Table 2) (23, 24, 44–60). They reported conflicting results on the effect of BTK deficiency on these cytokine productions upon TLR agonist stimulations (61), which may be due to the differences in experimental designs, including TLR agonists and BTK-deficient models used in these studies.

**Table 2:** Previous studies on the effect of BTK deficiency on cytokine productions in response to TLR agonists.

Cytokine	Effect of BTK deficiency on cytokine production	TLR tested	TLR agonist used	Cell type used	BTK-deficient model	Cytokine detection	Studies	Year
IFN-α	Decreased	TLR 9	ODN 2216	pDC	RN486	Flow cytometry	Wang et al. (44)	2014

	No effect	TLR 7/8	CL097/Loxoribine	pDC	XLA patient	ELISA	Marron et al. (45)	2012
			Gardiquimod	pDC	RN486	Flow cytometry	Wang et al. (44)	2014
	TLR 9	ODN-2006	pDC	XLA patient	ELISA	Cunningham-Rundles et al. (46)	2006

IFN-β	Decreased	TLR 3	Poly I: C	(Mice) BMDM	*BTK*−/− mice	ELISA	Lee et al. (23)	2012
		TLR 4	LPS	(Mice) BMDM	*BTK*−/− mice	ELISA	Lee et al. (23)	2012
		TLR 7/8	R848	(Mice) BMDC	*BTK*−/− mice	RT-PCR	Li et al. (47)	2014
		TLR 9	CpG-ODN1668	(Mice) BMDC	*BTK*−/− mice	RT-PCR/ELISA	Li et al. (47)	2014

Tumor necrosis factor alpha (TNF-α)	Decreased	TLR 2/6	PAM3Cys4	PBMC	XLA patient	ELISA	Horwood et al. (48)	2006
			PGN	MoDCs	XLA patient	ELISA	Taneichi et al. (49)	2008
		TLR 3	Poly I: C	MoDCs	XLA patient	ELISA	Taneichi et al. (49)	2008
				(Mice) BMDM	*BTK*−/− mice	ELISA	Lee et al. (23)	2012
		TLR 4	LPS	MoDCs	XLA patient	ELISA	Taneichi et al. (49)	2008
				Monocyte	XLA patient	ELISA	Horwood et al. (50)	2003
		TLR 7/8	R848	MoDCs	XLA patient	ELISA	Taneichi et al. (49)	2008
			ssRNA	MoDCs	XLA patient/LFM-A13	Flow cytometry	Sochorová et al. (51)	2007
		TLR 9	ODN-2216	MoDCs	XLA patient/Ibrutinib	RT-PCR	Lougaris et al. (52)	2014
				pDC	RN486	Flow cytometry	Wang et al. (44)	2014

	Increased	TLR 4	LPS	B cell depleted PBMC	XLA patient	ELISA	González-Serrano et al. (53)	2012
				Monocyte/MoDCs	XLA patient	Flow cytometry/ELISA	Marron et al. (45)	2012
		TLR 7/8	CL097	Monocyte/MoDCs	XLA patient	Flow cytometry/ELISA	Marron et al. (45)	2012

	No effect	TLR 2/6	PAM3Cys4	(Mice) BMDM	*BTK*−/− mice	ELISA	Köprülü et al. (54)	2013
			Zymosan	MoDCs	XLA patient/LFM-A13	Flow cytometry	Sochorová et al. (51)	2007
		TLR 3	Poly I: C	MoDCs	XLA patient/LFM-A13	Flow cytometry	Sochorová et al. (51)	2007
		TLR 4	LPS	MoDCs	XLA patient/LFM-A13	Flow cytometry	Sochorová et al. (51)	2007
				MoDCs	XLA patient/Ibrutinib	RT-PCR	Lougaris et al. (52)	2014
				Monocyte	XLA patient	Flow cytometry	de Diego et al. (55)	2006
		TLR 5	Flagellin	MoDCs	XLA patient/LFM-A13	Flow cytometry	Sochorová et al. (51)	2007
		TLR 7	Gardiquimod	pDC	RN486	Flow cytometry	Wang et al. (44)	2014
		TLR 9	ODN-2006	MoDCs	XLA patient	ELISA	Taneichi et al. (49)	2008

IL-1β	Decreased	TLR 2/6	PAM3Cys4	PBMC	XLA patient	ELISA	Horwood et al. (48)	2006
		TLR 4	LPS	PBMC	XLA patient	ELISA	Horwood et al. (48)	2006
		TLR 7	Imiquimod	(Mice) BMDM	*BTK*−/− mice	ECL assay	Byrne et al. (60)	2013

	Increased	TLR 4	LPS	B cell depleted PBMC	XLA patient	ELISA	González-Serrano et al. (53)	2012

IL-8	No effect	TLR 2/6	PAM3Cys4	PBMC	XLA patient	ELISA	Horwood et al. (48)	2006
		TLR 4	LPS	PBMC	XLA patient	ELISA	Horwood et al. (48)	2006

IL-10	Decreased	TLR 2/6	PGN	(Mice) BMDM	*BTK*−/− mice	ELISA	Schmidt et al. (56)	2006
		TLR 3	Poly I: C	(Mice) BMDM	*BTK*−/− mice	ELISA	Schmidt et al. (56)	2006
				(Mice) BMDM	*BTK*−/− mice	ELISA	Lee et al. (23)	2012
		TLR 4	LPS	(Mice) BMDM	*BTK*−/− mice	ELISA	Schmidt et al. (56)	2006
		TLR 7	Imiquimod	(Mice) BMDM	*BTK*−/− mice	ECL assay	Byrne et al. (60)	2013
		TLR 9	*E. coli* CpG	(Mice) BMDM	*BTK*−/− mice	ELISA	Schmidt et al. (56)	2006
			ODN-1826	(Mice) B lymphocyte	*BTK*−/− mice	ELISA	Lee et al. (57)	2008
	
	Increased	TLR 4	LPS	B cell depleted PBMC	XLA patient	ELISA	González-Serrano et al. (53)	2012
				MoDCs	XLA patient	ELISA	Gagliardi et al. (58)	2003
				(Mice) BMDM	*BTK*−/− mice	ELISA	Gabhann et al. (59)	2012
				MoDCs	XLA patient	ELISA	Marron et al. (45)	2012
		TLR 7/8	CL097	MoDCs	XLA patient	ELISA	Marron et al. (45)	2012
	
	No effect	TLR 2/6	PAM3Cys4	PBMC	XLA patient	ELISA	Horwood et al. (48)	2006
			Zymosan	MoDCs	XLA patient/LFM-A13	Flow cytometry	Sochorová et al. (51)	2007
		TLR 3	Poly I: C	MoDCs	XLA patient/LFM-A13	Flow cytometry	Sochorová et al. (51)	2007
		TLR 4	LPS	MoDCs	XLA patient/LFM-A13	Flow cytometry	Sochorová et al. (51)	2007
				PBMC	XLA patient	ELISA	Horwood et al. (48)	2006
				MoDCs	XLA patient/Ibrutinib	RT-PCR	Lougaris et al. (52)	2014
				Monocyte	XLA patient	ELISA	Marron et al. (45)	2012
		TLR 5	Flagellin	MoDCs	XLA patient/LFM-A13	Flow cytometry	Sochorová et al. (51)	2007
		TLR 7/8	CL097	Monocyte	XLA patient	ELISA	Marron et al. (45)	2012
			ssRNA	MoDCs	XLA patient/LFM-A13	Flow cytometry	Sochorová et al. (51)	2007
		TLR 9	ODN-2216	MoDCs	XLA patient/Ibrutinib	RT-PCR	Lougaris et al. (52)	2014

IL-6	Decreased	TLR 3	Poly I: C	(Mice) BMDM	*BTK*−/− mice	ELISA	Lee et al. (23)	2012
		TLR 8	ssRNA	MoDCs	XLA patient/LFM-A13	Flow cytometry	Sochorová et al. (51)	2007
		TLR 9	ODN-2216	MoDCs	XLA patient/Ibrutinib	RT-PCR	Lougaris et al. (52)	2014
				pDC	RN486	Flow cytometry	Wang et al. (44)	2014
			CpG-ODNs	PBMC	XLA patient/LFM-A13	ELISA	Doyle et al. (24)	2007

	Increased	TLR 4	LPS	B cell depleted PBMC	XLA patient	ELISA	González-Serrano et al. (53)	2012
				MoDCs	XLA patient	RT-PCR	Lougaris et al. (52)	2014
				(Mice) BMDM	*BTK*−/− mice	ELISA	Schmidt et al. (56)	2006
				Monocyte/MoDCs	XLA patient	Flow cytometry/ELISA	Marron et al. (45)	2012
		TLR 7/8	CL097	Monocyte/MoDCs	XLA patient	Flow cytometry/ELISA	Marron et al. (45)	2012
		TLR 9	ODN-1826	(Mice) B lymphocyte	*BTK*−/− mice	ELISA	Lee et al. (57)	2008

	No effect	TLR 2/6	PAM3Cys4	PBMC	XLA patient	ELISA	Horwood et al. (48)	2006
				(Mice) BMDM	*BTK*−/− mice	ELISA	Köprülü et al. (54)	2013
			Zymosan	MoDCs	XLA patient/LFM-A13	Flow cytometry	Sochorová et al. (51)	2007
			PGN	MoDCs	XLA patient	ELISA	Taneichi et al. (49)	2008
		TLR 3	Poly I: C	MoDCs	XLA patient	ELISA	Taneichi et al. (49)	2008
				MoDCs	XLA patient/LFM-A13	Flow cytometry	Sochorová et al. (51)	2007
		TLR 4	LPS	MoDCs	XLA patient/LFM-A13	Flow cytometry	Sochorová et al. (51)	2007
				PBMC	XLA patient	ELISA	Horwood et al. (48)	2006
				MoDCs	XLA patient	ELISA	Taneichi et al. (49)	2008
				MoDCs	Ibrutinib	RT-PCR	Lougaris et al. (52)	2014
				Monocyte	XLA patient	Flow cytometry	de Diego et al. (55)	2006
		TLR 5	Flagellin	MoDCs	XLA patient/LFM-A13	Flow cytometry	Sochorová et al. (51)	2007
		TLR 7/8	R848	MoDCs	XLA patient	ELISA	Taneichi et al. (49)	2008
			Gardiquimod	pDC	RN486	Flow cytometry	Wang et al. (44)	2014
		TLR 9	ODN-2006	MoDCs	XLA patient	ELISA	Taneichi et al. (49)	2008

IL-12	Decreased	TLR 3	Poly I:C	(Mice) BMDM	*BTK*−/− mice	ELISA	Lee et al. (23)	2012
		TLR 4	LPS	(Mice) BMDM	*BTK*−/− mice	ELISA	Gabhann et al. (59)	2012
		TLR 7	Imiquimod	(Mice) BMDM	*BTK*−/− mice	ECL assay	Byrne et al. (60)	2013
		TLR 9	ODN-2216	MoDCs	XLA patient/Ibrutinib	RT-PCR	Lougaris et al. (52)	2014

	Increased	TLR 9	ODN-1826	(Mice) B lymphocyte	*BTK*−/− mice	ELISA	Lee et al. (57)	2008
	
	No effect	TLR 2/6	Zymosan	MoDCs	XLA patient/LFM-A13	Flow cytometry	Sochorová et al. (51)	2007
		PGN	MoDCs	XLA patient	ELISA	Taneichi et al. (49)	2008
		TLR 3	Poly I:C	MoDCs	XLA patient/LFM-A13	Flow cytometry	Sochorová et al. (51)	2007
				MoDCs	XLA patient	ELISA	Taneichi et al. (49)	2008
		TLR 4	LPS	MoDCs	XLA patient/LFM-A13	Flow cytometry	Sochorová et al. (51)	2007
				MoDCs	XLA patient	ELISA	Taneichi et al. (49)	2008
				MoDCs	XLA patient	ELISA	Gagliardi et al. (58)	2003
				MoDCs	XLA patient/Ibrutinib	RT-PCR	Lougaris et al. (52)	2014
		TLR 5	Flagellin	MoDCs	XLA patient/LFM-A13	Flow cytometry	Sochorová et al. (51)	2007
		TLR 7/8	R848	MoDCs	XLA patient	ELISA	Taneichi et al. (49)	2008
			ssRNA	MoDCs	XLA patient/LFM-A13	Flow cytometry	Sochorová et al. (51)	2007
		TLR 9	ODN-2006	MoDCs	XLA patient	ELISA	Taneichi et al. (49)	2008

IL-18	Decreased	TLR 4	LPS	(Mice) BMDM	*BTK*−/− mice	ELISA	Gabhann et al. (59)	2012

CXCL-1	Decreased	TLR 7	Imiquimod	(Mice) BMDM	*BTK*−/− mice	ECL assay	Byrne et al. (60)	2013

*PGN, Zymosan and PAM3Cys4 are TLR 2/6 agonist. Poly I:C is TLR 3 agonist. LPS is TLR4 agonist. Flagellin is TLR5 agonist. CL097 and R848 are TLR 7/8 agonist. Gardiquimod and Loxoribine are TLR 7 agoinst. ssRNA is TLR8 agonist. E. coli CpG, CpG-ODNs, ODN-2006, CpG-ODN1668, ODN-1826, and ODN-2216 are TLR9 agonist. LFM-A13, RN486, and Ibrutinib are BTK inhibitors*.

*TLR, toll-like receptor; IFN, interferon; IL, interleukin; CXCL, chemokine (C-X-C motif) ligand; ECL assay, electrochemiluminescence assay; pDC, plasmacytoid dendritic cells; BMDM, bone marrow-derived macrophages; BMDC, bone marrow-derived conventional dendritic cells; MoDCs, monocyte-derived dendritic cells; PBMC, peripheral blood mononuclear cells; XLA, X-linked agammaglobulinemia; BTK, Bruton’s tyrosine kinase*.

In our current study, using OPV instead of TLR agonists as stimulation resulted in more complex interactions between virus and host, as OPV can be recognized by various PRRs. To date there are at least 3 more studies in addition to our current study that used pathogen instead of TLR agonists to stimulate cytokine productions (Table 3) (4, 23, 54). Cytokine productions in response to live or dead pathogen stimulation are different (54) as live pathogen may have mechanisms to evade immunity (62, 63), which further complicate the virus–host interactions.

**Table 3:** Studies of the effect of BTK deficiency on cytokine productions in response to pathogen stimulation.

Cytokine	Effect of BTK-deficiency on cytokine production	Pathogen used	Cell used	BTK-deficiency model	Cytokine detection	Studies	Year
Type I IFN	Decreased	OPV	MoDCs	XLA patients/LFM-A13	Flow cytometry	Current study	2018
		Dengue virus	BMDM	*BTK*−/− mice	Semiquantitative PCR	Lee et al. (23)	2012

	No effect	H1N1	MoDCs	XLA patients/LFM-A13	Flow cytometry	Current study	2018
			MoDCs	XLA patients	ELISA	Liu et al. (4)	2012
		*Listeria monocytogenes*	BMDM	*BTK*−/− mice	Bioassay	Köprülü et al. (54)	2013

Type III IFN	Decreased	OPV	MoDCs	XLA patients/LFM-A13	Flow cytometry	Current study	2018

IP-10	Decreased	OPV	MoDCs	XLA patients	Flow cytometry	Current study	2018
	No effect	OPV	MoDCs	LFM-A13	Flow cytometry	Current study	2018
		H1N1	MoDCs	XLA patients/LFM-A13	Flow cytometry	Current study	2018

IL-1β	No effect	OPV	MoDCs	XLA patients/LFM-A13	Flow cytometry	Current study	2018
		H1N1	MoDCs	XLA patients/LFM-A13	Flow cytometry	Current study	2018

TNF-α	Increased	*L. monocytogenes*	BMDM	*BTK*−/− mice	ELISA	Köprülü et al. (54)	2013
	No effect	OPV	MoDCs	XLA patients/LFM-A13	Flow cytometry	Current study	2018
		H1N1	MoDCs	XLA patients/LFM-A13	Flow cytometry	Current study	2018
		(Heat killed) *L. monocytogenes*	BMDM	*BTK*−/− mice	ELISA	Köprülü et al. (54)	2013

IL-8	No effect	OPV	MoDCs	XLA patients/LFM-A13	Flow cytometry	Current study	2018
		H1N1	MoDCs	XLA patients/LFM-A13	Flow cytometry	Current study	2018

IL-6	Increased	*L. monocytogenes*	BMDM	*BTK*−/− mice	ELISA	Köprülü et al. (54)	2013
	No effect	(Heat killed) *L. monocytogenes*	BMDM	*BTK*−/− mice	ELISA	Köprülü et al. (54)	2013
IL-10	No effect	*L. monocytogenes*	BMDM	*BTK*−/− mice	ELISA	Köprülü et al. (54)	2013

IL-12	Increased	*L. monocytogenes*	BMDM	*BTK*−/− mice	Flow cytometry (IL12p40)	Köprülü et al. (54)	2013
	No effect	*L. monocytogenes*	BMDM	*BTK*−/− mice	ELISA (IL12p70)	Köprülü et al. (54)	2013
		H1N1	MoDCs	XLA patients	ELISA	Liu et al. (4)	2012

*LFM-A13 is a BTK inhibitor. IFN, interferon; IP-10, interferon gamma-induced protein 10; IL, interleukin; OPV, oral poliovirus vaccine Sabine 1; MoDCs, monocyte-derived dendritic cells; BMDM, bone marrow-derived macrophages; XLA, X-linked agammaglobulinemia; BTK, Bruton’s tyrosine kinase*.

In our current study, we used XLA patients and LFM-A13 inhibited healthy control as BTK-deficiency groups. LFM-A13 has been used to generate BTK-deficiency models in previous studies (24, 51, 60), along with Ibrutinib and RN486 (Table 2) (44, 52). LFM-A13 is a dual kinase inhibitor for BTK and Polo-like kinase 3 (PLK3) (64, 65). PLK3 is a serine/threonine protein kinase that regulates cell cycle and apoptosis (66). Since PLK3 is not involved in TLRs signaling or IFN productions (67), therefore using LFM-A13 in our current study would demonstrate the impairment of type I and III IFN productions were through BTK inhibition.

Phenotypic maturation of MoDCs, characterized by increased expressions of CD83, CD86, and MHC-II, was impaired in MoDCs of XLA patients upon OPV but not H1N1 stimulation. However, such pattern was not demonstrated by inhibiting BTK in MoDCs of healthy controls. It has been shown that MoDCs of XLA patients have partial impairment of phenotypic maturation when stimulated with TLRs agonists (49). Such partial impairment could be explained by the maturation of dendritic cells not only depends on TLR engagement but also on RLRs, type I IFN, and TNF-α (68–70).

There was no difference in the rise of OPV RNA between MoDCs of XLA patients and healthy controls in spite of the near absence of type I and III IFN productions in MoDCs of XLA patients. With the use of MOI of 1 during viral stimulation, all MoDCs were simultaneously exposed to OPV before type I and III IFN were produced to limit OPV replication. In addition, with the replication capacity of OPV attenuated (71), the protection effect of IFN may not be critical, resulting in no difference in replication between the two groups. More importantly, the lack of difference in OPV RNA level indicated the difference in type I and III IFN productions between MoDCs of XLA patients and healthy controls were independent of the titers of OPV.

All patients in our current study received OPV and none developed complications, namely VAPP and VDPV excretion (Table 1) (5, 6, 72). The risk of VAPP in the world is approximately four cases per million births (73). Patients with antibody deficiencies, including XLA patients, are 3,000 to 10,000 times more susceptible to have VAPP compared to general population (8, 9) (i.e., with a risk of 1.2–4%). In addition, XLA patients can have prolonged VDPV excretion for as long as 5 years (5), thus posing a significant obstacle for global eradication of poliovirus.

Apart from antibody deficiency, impaired type I IFN response has been hypothesized before to be responsible for developing VAPP in general population by allowing unchecked multiplication of OPV in extraneural tissues (74). However, IFN or antibody deficiency alone cannot explain the susceptibility of having VAPP and VDPV excretion in XLA patients. Hyper IgM syndrome patients, who have a deficiency of IgG, IgA, and IgE, are not susceptible to VAPP or VDPV excretion. In addition, they are less susceptible to severe enterovirus infections compared to XLA patients (75). Similarly, IFN deficiency patients, including those with TLR3 and UNC-93B deficiency, are not susceptible to OPV vaccination complications or severe enterovirus infections (76, 77). Therefore, it may require a combined impairment of both IFN and antibody to render XLA patients susceptible toward severe enterovirus infections.

**Figure 8:**
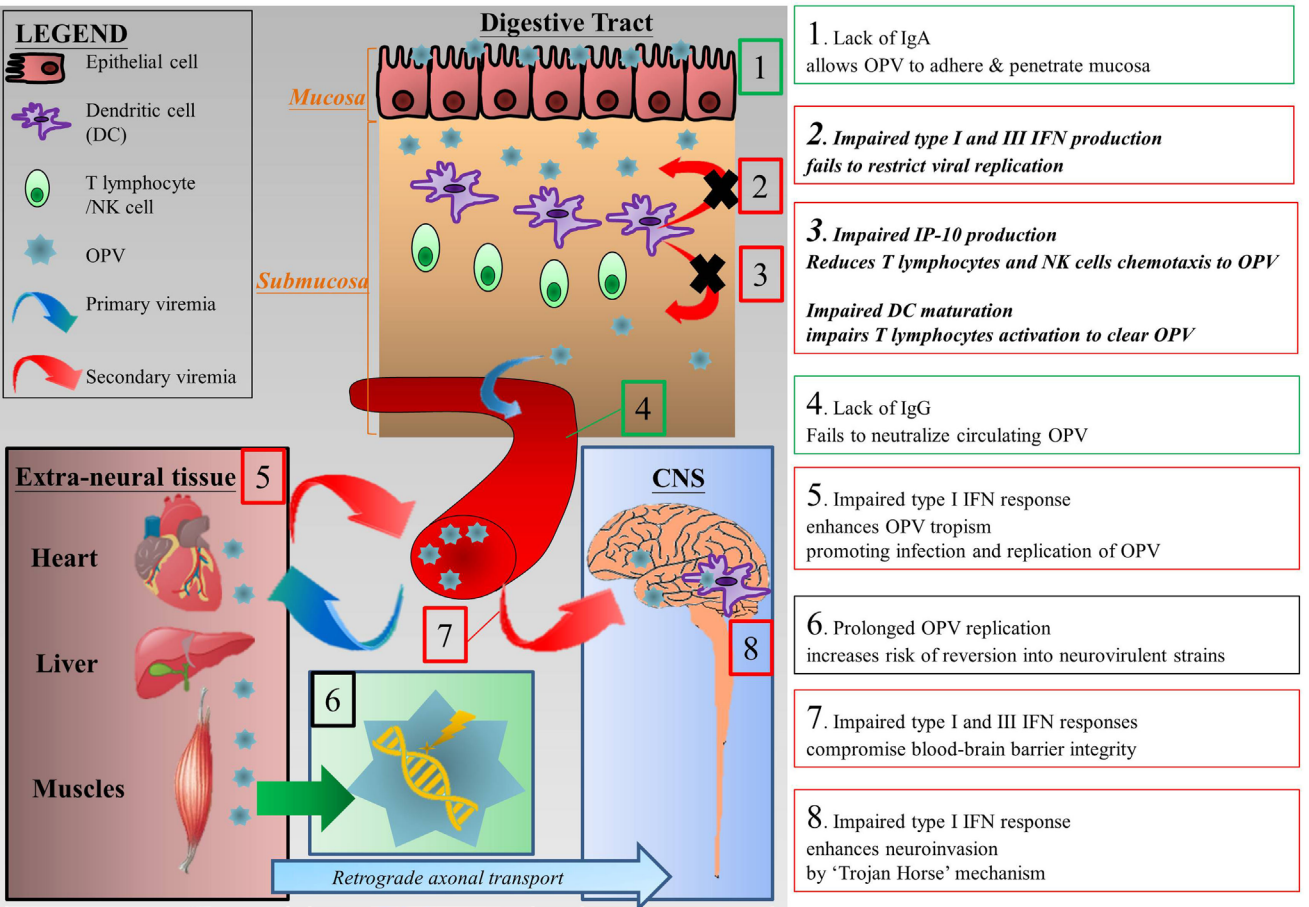
Postulated mechanisms that increase the risk of vaccine-associated paralytic poliomyelitis in XLA patients. (1) Lack of secretory Ig A allows OPV to adhere and penetrate digestive tract mucosa (78). (2) At the mucosa, restriction of viral replication is impaired due to impaired type I and III IFN productions (14, 15). (3) In addition, impaired IP-10 production and phenotypic maturation of dendritic cells reduce T lymphocytes migration and activation which in turns impair clearance of OPV infection (42, 43). (4) The lack of neutralizing antibodies in XLA patients results in prolonged viremia. (5) The lack of type I IFN enhances tropism of OPV, allowing more extensive infection of extraneural tissues by OPV and thus enhancing replication of OPV, which leads to sustained secondary viremia (74, 80). (6) Prolonged replication of OPV increases the chance of mutation which may revert the vaccine strain to a neurovirulent one (5, 6, 73, 81). (7) Type III IFN protects the epithelium component of blood–brain barrier, while type I IFN protects the endothelium component. Combined impairment of both type I and III IFN compromises blood–brain barrier integrity (82). (8) Finally, OPV can enter central nervous system (CNS) *via* “Trojan horse” mechanism in which they cross the blood–brain barrier by hiding in immune cells such as macrophages. Type I IFN response may limit CNS invasion by “Trojan horse” mechanism (82). Green textboxes represent humoral immunodeficiency and its consequences in XLA patients and red textboxes represent innate immunodeficiencies and their consequences in XLA patients. Textboxes with italic and bolded text represent our findings and their consequences. Abbreviations: CNS, central nervous system; IFN, interferon; IP-10, interferon gamma-induced protein 10; OPV, oral polio vaccine.

Therefore, based on our findings and previous reports, we postulated the increased risk of VAPP in XLA patients is due to combined deficiency of IFN and antibody which impairs defenses at various checkpoints against the development of VAPP after OPV vaccination (Figure 8). The lack of secretory Ig A on mucosa promotes the adherence and invasion of OPV (78). Decreased type I and III IFN and impaired maturation of antigen presentation cells may permit unrestricted viral replication and impair T cell recruitment as well as activation (14, 15). In addition, our previous study has shown that BTK-deficient NK cells have impaired IFN-γ production and reduced cytotoxicity in response to TLR3 stimulation (79). Together these defects result in prolonged replication and shedding of OPV. Moreover, decreased type I IFN and antibody deficiency result in enhanced tissue tropism of OPV which may lead to more effective infection of extraneural tissue and OPV replication (74, 80). Defects in these checkpoints allow OPV replication which may result in reversion of neurovirulence through mutations, thus increasing risk of having VAPP and excreting VDPV (5, 6, 73, 81). Since type I and III IFN offers some protection against CNS invasion by maintaining the integrity of blood–brain barrier (82), the integrity of blood–brain barrier in XLA patients may be compromised, becoming more vulnerable to neuroinvasion.

In conclusion, selective impairment of type I and III IFN productions in response to OPV but not to H1N1 was demonstrated in XLA patients, implicating BTK-dependent impairments are responsible for the unique susceptibility of these patients to severe enterovirus infections.

## Ethics Statement

This study was carried out in accordance with the recommendations of the Institutional Review Board of the University of Hong Kong/Hospital Authority Hong Kong West Cluster (UW 08-002) with written informed consent from all subjects. All subjects gave written informed consent in accordance with the Declaration of Helsinki. The protocol was approved by the Institutional Review Board of the University of Hong Kong/Hospital Authority Hong Kong West Cluster (UW 08-002).

## Author Contributions

WT and YL designed the study and discussed with HM who then wrote the research proposal. PL and YL recruited the patients for this study. AL, KN, YW, KL, and KC performed the experiments. WT and YL supervised the experiments and interpreted the data. AL and YL wrote the manuscript with extensive appraisal from PL, WT, and HM.

## Conflict of Interest Statement

The authors declare that the research was conducted in the absence of any commercial or financial relationships that could be construed as a potential conflict of interest.

## Supplementary Material

Figure S1 IFN-β production in MoDCs from healthy controls (*n* = 4) and Bruton’s tyrosine kinase-inhibited MoDCs from healthy controls (*n* = 4) upon OPV and H1N1 stimulation. LFM-A13 at 0, 100, and 150 µM were added to MoDCs from healthy controls 2 h prior to viral stimulation. Data represented as mean + SEM. **p* < 0.05; ns, *p* > 0.05.

Figure S2 IL-1β, tumor necrosis factor alpha, and IL-8 productions in MoDCs from healthy controls (*n* = 23), Bruton’s tyrosine kinase (BTK)-inhibited MoDCs from healthy controls (*n* = 11), and MoDCs from XLA patients (*n* = 9) upon OPV stimulation. MoDCs were stimulated with OPV at multiplicity of infection of 1 for 24 h. Open symbols represent MoDCs stimulated with OPV; filled symbols represent MoDCs that were mock-stimulated with RPMI. Healthy + INH, BTK-inhibited MoDCs from healthy controls. Data represented as mean ± SEM.

Figure S3 IL-1β, tumor necrosis factor alpha, and IL-8 productions in MoDCs from healthy controls (*n* = 15), Bruton’s tyrosine kinase (BTK)-inhibited MoDCs from healthy controls (*n* = 9), and MoDCs from XLA patients (*n* = 9) upon H1N1 stimulation. MoDCs were stimulated with H1N1 at multiplicity of infection of 1 for 24 h. Open symbols represent MoDCs stimulated with H1N1; filled symbols represent MoDCs that were mock-stimulated with RPMI. Controls of healthy controls, BTK-inhibited healthy controls, and XLA patients were subsets of controls in Figure S2 in Supplementary Material. Healthy + INH, BTK-inhibited MoDCs from healthy controls. Data represented as mean ± SEM.

## Abbreviations

BMDC, bone marrow-derived conventional dendritic cells; BMDM, bone marrow-derived macrophages; BTK, Bruton’s tyrosine kinase; CNS, central nervous system; CXCL, chemokine (C-X-C motif) ligand; MoDCs, monocyte-derived dendritic cells; ECL, electrochemiluminescence; FBS, fetal bovine serum; GAPDH, glyceraldehyde-3-phosphate dehydrogenase; IFN, interferon; IL, interleukin; IP-10, interferon gamma-induced protein 10; IRF, interferon regulatory factor; IVIG, intravenous immunoglobulin; MDA5, melanoma differentiation-associated protein 5; MFI, mean fluorescent intensity; MOI, multiplicity of infection; MX1, MX dynamin like GTPase 1; MyD88, myeloid differentiation primary response gene 88; NK, natural killer; OAS1, 2′-5′ oligoadenylate synthetase 1; OPV, oral poliovirus vaccine; PAMPs, pathogen-associated molecular patterns; PBMC, peripheral blood mononuclear cells; pDCs, plasmacytoid dendritic cells; PH, pleckstrin homology; PID, primary immunodeficiency; PK, protein kinase; PKR, protein kinase R; PLK3, Polo-like kinase 3; PRRs, pattern recognition receptors; RIG-I, retinoic acid-inducible gene I; RLRs, RIG-I-like receptors; SEM, standard error of mean; SH2, Src homology 2; TIR, toll/interleukin-1 receptor; TIRAP, TIR domain-containing adaptor protein; TLRs, toll-like receptors; TNF-α, tumor necrosis factor alpha; TRIF, TIR-domain-containing adapter-inducing interferon-β; VAPP, vaccine-associated paralytic poliomyelitis; VDPV, vaccine-derived poliovirus; XLA, X-linked agammaglobulinemia.
