# Supplementary figures and images for: Type I and III Interferon Productions Are Impaired in X-Linked Agammaglobulinemia Patients Toward Poliovirus but Not Influenza Virus
Source: Front Immunol. 2018 Aug 10;9:1826. doi: 10.3389/fimmu.2018.01826 (PMC6095995; doi:10.3389/fimmu.2018.01826)

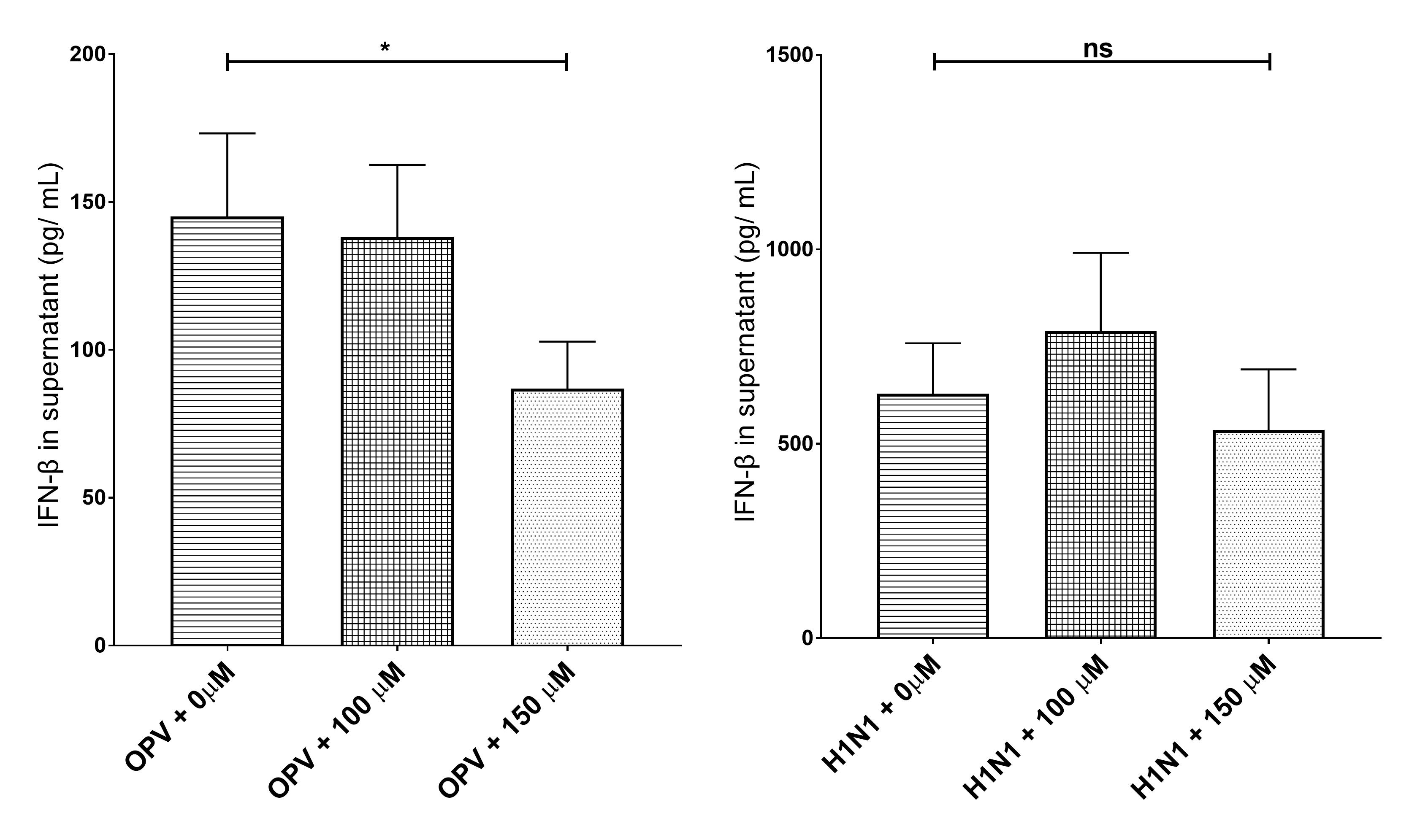

Supplement: Figure S1 — IFN-β production in MoDCs from healthy controls (n = 4) and Bruton’s tyrosine kinase-inhibited MoDCs from healthy controls (n = 4) upon OPV and H1N1 stimulation. LFM-A13 at 0, 100, and 150 µM were added to MoDCs from healthy controls 2 h prior to viral stimulation. Data represented as mean + SEM. *p < 0.05; ns, p > 0.05. [file image_1.jpeg]

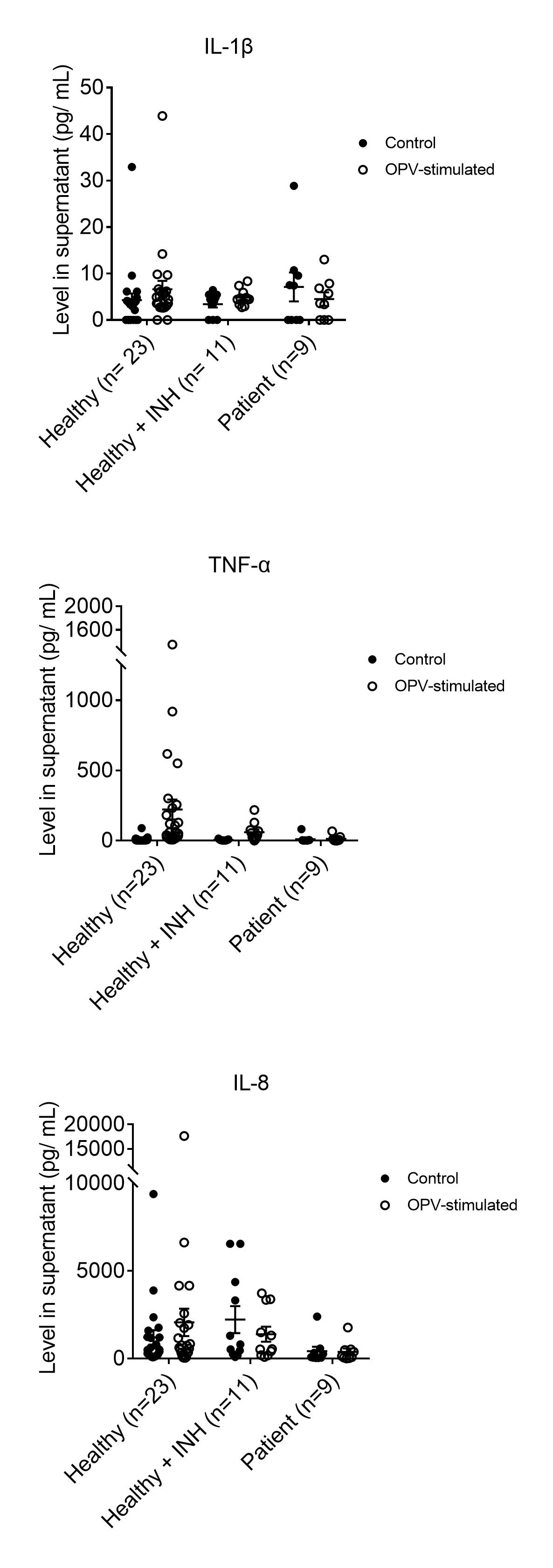

Supplement: Figure S2 — IL-1β, tumor necrosis factor alpha, and IL-8 productions in MoDCs from healthy controls (n = 23), Bruton’s tyrosine kinase (BTK)-inhibited MoDCs from healthy controls (n = 11), and MoDCs from XLA patients (n = 9) upon OPV stimulation. MoDCs were stimulated with OPV at multiplicity of infection of 1 for 24 h. Open symbols represent MoDCs stimulated with OPV; filled symbols represent MoDCs that were mock-stimulated with RPMI. Healthy + INH, BTK-inhibited MoDCs from healthy controls. Data represented as mean ± SEM. [file image_2.jpeg]

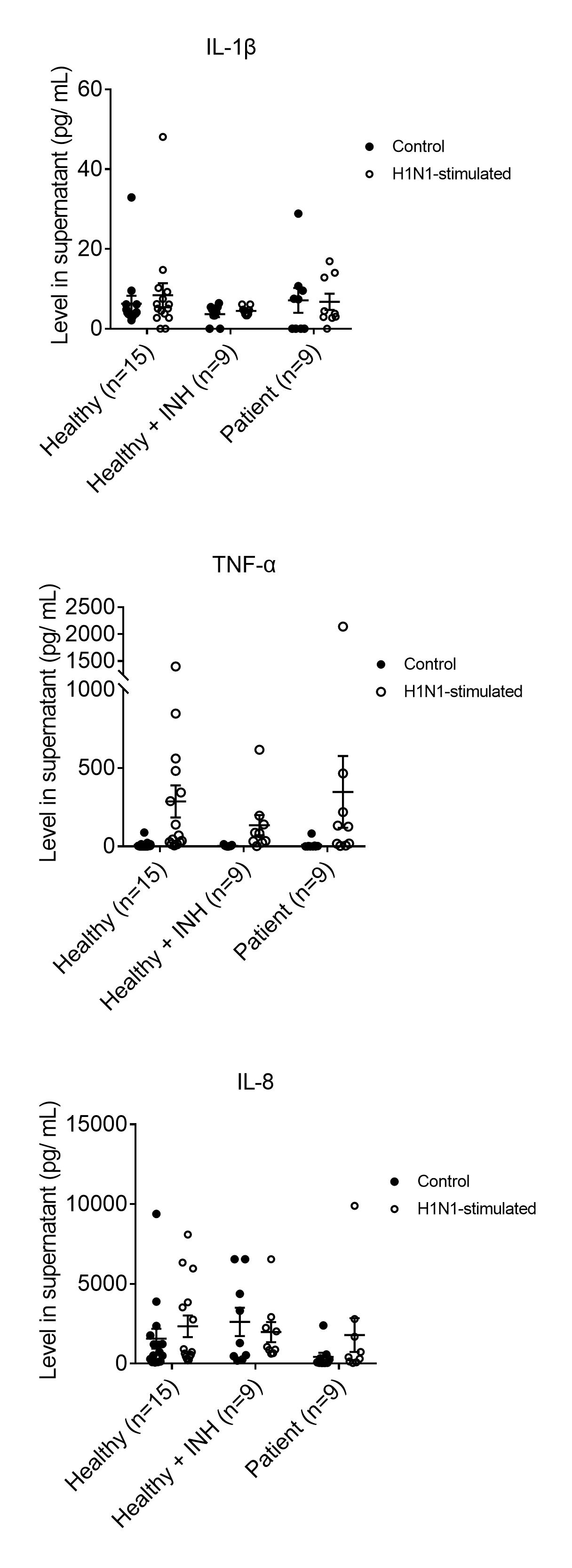

Supplement: Figure S3 — IL-1β, tumor necrosis factor alpha, and IL-8 productions in MoDCs from healthy controls (n = 15), Bruton’s tyrosine kinase (BTK)-inhibited MoDCs from healthy controls (n = 9), and MoDCs from XLA patients (n = 9) upon H1N1 stimulation. MoDCs were stimulated with H1N1 at multiplicity of infection of 1 for 24 h. Open symbols represent MoDCs stimulated with H1N1; filled symbols represent MoDCs that were mock-stimulated with RPMI. Controls of healthy controls, BTK-inhibited healthy controls, and XLA patients were subsets of controls in Figure S2 in Supplementary Material. Healthy + INH, BTK-inhibited MoDCs from healthy controls. Data represented as mean ± SEM. [file image_3.jpeg]
